# Growth and maturity status of female table tennis players seeking authorization to compete in older age categories

**DOI:** 10.1186/s13102-026-01903-6

**Published:** 2026-07-30

**Authors:** Tomas Oliveira, Sylwia Bartkowiak, Jan M. Konarski, Magdalena Krzykała, Oscar M. Tavares, Rute Santos, Tiago D. M. Patrão, Inês Rodrigues, Jorge M. Celis-Moreno, João Valente-dos-Santos, Ricardo R. Agostinete, Rômulo Araújo Fernandes, Enio R. V. Ronque, Diogo V. Martinho, Manuel J. Coelho-e-Silva, Robert M. Malina

**Affiliations:** 1 Physical Education and Sport Center, Londrina State University, Londrina, Paraná Brazil; 2 Londrina State University, GEPEMENE (Edital 1, 2026), Londrina, Paraná Brazil; 3 Theory of Sports Department (Sport Science), Poznań University of Physical Education, Poznań, Poland; 4 Department of Methodology of Recreation, Poznań University of Physical Education, Poznań, Poland; 5 Polytechnic Institute of Coimbra, Coimbra Health School, Coimbra, Portugal; 6 SANFIL, Coimbra, Portugal; 7 Portuguese Institute of Sports and Youth, Sports Medicine Center, Porto, Portugal; 8 University of Coimbra, FCDEF, Pavilhão III, Coimbra, 3040-156 Portugal; 9 University of Santo Tomas, Bogota, Colombia; 10 Universidade Lusófona, CIDEFES, Lisbon, Portugal; 11 Sporting Clube Portugal, COD (Center of Sports Optimization), Lisbon, Portugal; 12 Department of Physical Education (Laboratory of Investigation in Exercise - LIVE), Sao Paulo State University (UNESP), Presidente Prudente, Sao Paulo, Brazil; 13 University of Coimbra, CIPER, Coimbra, Portugal; 14 Department of Kinesiology and Health Education, University of Texas at Austin, Austin, TX United States of America; 15 School of Public Health and Information Sciences, University of Louisville, Louisville, KY United States of America

**Keywords:** Growth status, Skeletal age, Youth athletes, Adolescence

## Abstract

**Background:**

Given the relatively small number of female youth table tennis players in some competitive age categories in Portugal, participants increasingly request authorization to compete in older age groups. Therefore, the aim of this study is to examine the growth and skeletal maturity status, and the body composition of female table tennis players in three competitive chronological age (CA) groups: U11, U13 and U15.

**Methods:**

The sample included 61 female table tennis players 9.0 to 16.4 years who underwent medical evaluation associated with requests to compete in CA categories. The players were classified as U11 (*n* = 13), U13 (*n* = 26), and U15 (*n* = 21) Variables included stature, body mass, skeletal age (SA) using Fels method, triceps and subscapular skinfolds, and estimates of fat mass and fat free mass. Based on SA minus CA), players were classified as late, on time, or early maturing. Characteristics were examined by competitive CA group and maturity status.

**Results:**

Statures and weights of players ranged from < 10th to > 90th percentiles of the respective references, while means by competitive CA groups were generally above the respective reference medians. The trend in body size was consistent with advanced skeletal maturity status as SA was in advance of CA by approximately one year, on average, within each CA group. Fat-free mass increased across CA groups, while percentage fat mass was relatively stable at 20–22%. Maturity-related variation was considerable within and among the competitive CA groups.

**Conclusion:**

Variation in growth and skeletal maturity status and in body composition of female table tennis players was considerable within and overlapped between adjacent competitive CA groups. Variation in growth and maturity status among youth players has implications for talent identification and training. Assessment of skeletal maturity status can facilitate the individualization of developmentally appropriate approaches in youth sports.

**Supplementary Information:**

The online version contains supplementary material available at https://doi.org/10.1186/s13102-026-01903-6.

## Introduction

Approximately 40 million individuals who regularly train and compete in table tennis worldwide are registered with national federations affiliated with the International Table Tennis Federation [1]. Match performance in table tennis is characterized by repetitive short-term efforts of high intensity during a rally interspersed with recovery bouts [2]. Although the anaerobic system tends to be the prominent energetic system during matches in table tennis and other racket sports, the competitive system also requires aerobic fitness largely in the context of recovery for the next match [3]. Recognizing the combination of complex motor skills and functional capacities in addition to mental and tactical skills associated with table tennis, the *Netherlands Table Tennis Federation* adopted a battery of tests in their talent development program [4, 5]. The battery consisted of tests associated with the ability for quick accelerations and turns and for quick and simultaneous coordinated arm and leg movements, speed in controlling a ball during sideward zigzag movements, eye–hand coordination, and the vertical jump.

Although the importance of anthropometric, performance and motor coordination characteristics of players were highly ranked by coaches of three racket sports – tennis, badminton and table tennis [1], the literature addressing the growth and maturity characteristics of youth table tennis players is quite limited. A recent study of the growth and skeletal maturity status of Portuguese and Polish male table tennis players aged 10 through 14 years [6] noted that the players tended to have heights and weights above the respective U.S. reference medians, and skeletal ages (SA) that were in advance of chronological ages (CA). Among elite British participants of both sexes 8–17 years in regular tennis, means for stature and body mass fluctuated close to the 75th percentiles of the respective U.S. reference data though trends in SA varied among the female players [7], while among elite Dutch male players 10–15 years, CA, body size and lower limb explosive strength were significant longitudinal predictors of 5-m sprint time [8]. Allowing for the limited data for youth table tennis, variation in the growth and maturity status in the respective samples was considerable and consistent with corresponding observations of youth participants in a variety of sports [9].

Competitive groups for participants in table tennis are organized by CA: under-11 (U11), under-13 (U13), under-15 (U15), under-19 (U19), under-21 (U21), and seniors [10]. Of interest, the U19 group spans four chronological years compared to two-year ranges in younger CA groups as well as in many other sports. The preceding applies to both sexes and may reflect limited opportunities for success among late adolescent table tennis players. It has also been noted that a better fitness level was associated with a more offensive and high intensity style of play in table tennis [11]. In the context of the preceding, CA- and maturity-associated variation in body size and fitness of table tennis participants merits attention as it may influence success in the sport.

The CA based competitive structure of table tennis applies both to males and females and does not take into consideration the sex differences in the timing and tempo of the pubertal growth spurt. Although technical, tactical, perceptual motor, and physical characteristics all contribute to performance in table tennis [11], considerable variation in growth and biological maturation likely exists among athletes within the same competitive CA group. As such, study of the growth and maturity status of youth table tennis players may contribute to a better understanding of developmental differences. Given the limited number of female participants in younger competitive categories in Portugal, it is relevant that a substantial number of adolescent female athletes in table tennis and several other sports have requested permission to train and compete in older CA groups [12]. “Moving up” to older CA groups in Portugal requires a special medical examination [13] which includes stature, body mass, skinfolds, echocardiography and SA based on a radiograph of the left hand and wrist.

Given the limited participation of Portuguese youth in table tennis, particularly females, studies addressing growth, biological maturation and body composition are needed. Thus, the objective of this study is to examine the growth and skeletal maturity status, and the body composition of female table tennis players in three competitive CA groups, U11, U13 and U15. Such information may inform future discussions regarding age group structure in female table tennis, particularly in the context of the earlier timing of pubertal growth in girls compared with boys.

## Materials and methods

### Procedures and ethics

Following an agreement between the *University of Coimbra* and the *Portuguese Institute of Sports* (IPDJ/FCDEF.UC/2017-01), the research project was approved by the *Ethics Committee* of the *Faculty of Sport Sciences and Physical Education* at the *University of Coimbra* (CE/FCDEF-UC/00122014). In Portugal, participants are legally able to provide informed consent upon reaching 18 years of age. In the present study, all participants were under the preceding limit at the time of assessment. Consequently, written informed consent was obtained from their parents or legal guardians during their visit to the *Center for Sports Medicine*, as part of the mandatory medical examination required for young athletes applying for authorization to compete in older age categories. The participants also read the consent form and were verbally informed of potential benefits and risks of the procedures, as well as the voluntary nature of their participation.

### Participants

All participants were registered with the *Portuguese Table Tennis Federation*. Inclusion criteria included: (i) at least two years of training experience in competitive table tennis; and (ii) a request to participate and compete in older competitive categories defined by CA as follows U11, U13, U15, U19, U21, and seniors [10]. Although the sample included 61 participants aged 9.03–16.43 years (CA was the difference between date of the clinical examination and date of birth), the oldest player exceeded the range of the U15 sample and was not considered in the specific analysis of players in the U11, U13 and U15 competitive CA groups. All players trained in 2–4 sessions per week (~ 90–120 min) and usually competed on weekends. The clubs participated in a 9-month regional competitive season (September - May) through the Portuguese Federation.

### Skeletal age

SA was estimated from radiograph of the left hand and wrist using the Fels method [14]. The protocol considers specific indicators for the radius, ulna, capitate, hamate, triquetral, pisiform, lunate, scaphoid, trapezium, trapezoid, adductor sesamoid, metacarpals I, III and V, proximal phalanges I, III and V, medial phalanges III and V, and distal phalanges I, III and V. Maturity grades for each bone include the presence or absence of the center of ossification in the radiograph, changes in shape, ratios of epiphyseal and diaphyseal widths of the long bones, and capping and fusion of the long bones. Assigned grades and measurements were entered into the Felshw 1.0 software (Felshw 1.0, Software Lifespan Health and Research Center, Departments of Community Health and Pediatrics, Boonshoft School of Medicine, Wright State University, Dayton, Ohio, USA) to obtain an estimate of SA and its standard error. All radiographs were assessed by a single experienced observer who had completed more than 1000 skeletal age assessments using the Fels method and had previously participated in studies addressing the intra observer reproducibility and inter observer agreement of Fels skeletal age assessments [15].

### Body size

Anthropometric dimensions were measured using standard procedures. Stature and sitting height were measured to the nearest 0.1 cm using a portable stadiometer (Harpenden model 98.603, Holtain Ltd, Crosswell, UK) and sitting height table (Harpenden sitting height table, model 98.607, Holtain Ltd. Crosswell, UK), respectively. Leg length was estimated as stature minus sitting height. Body mass was measured to the nearest 0.1 kg with a SECA balance (model 770, Hanover, MD, USA). The triceps and subscapular skinfold thicknesses were measured to the nearest mm with a Lange caliper (Beta Technology, Ann Arbor, MI, USA). All variables were measured on two separate occasions for a subsample of 13 players to estimate intra-observer technical errors of measurement [16]: stature (0.37 cm), sitting height (0.71 cm), body mass (0.56 kg) and skinfolds (0.47–0.72 mm). The errors were within the range of intra- and inter-observer errors in several surveys in the United States and a variety of field surveys, including studies of young athletes [17]. Body fat percentage (FM%) was predicted from the triceps and subscapular skinfolds using an equation proposed for female adolescents of White/European ancestry [18]. Fat mass (FM) and fat-free mass (FFM) were subsequently calculated.

### Analyses

Descriptive statistics were calculated for the total sample of 61 players (minimum, maximum, means, standard deviations) for CA, SA, SA-to-CA ratio, SA minus CA, anthropometric and derived variables. The stature and body mass of individual table tennis players were also plotted by CA relative to percentiles of the respective US references [19] to illustrate variation in growth status in the sample. Mean heights and weights in the three competitive CA groups were also plotted relative to the US references. The oldest player in the sample, aged 16.43 years, was not included in the analysis of players in the three competitive CA groups. Fels SAs were plotted relative to CAs for individual players and as mean SAs (± standard deviation) relative to mean CAs (± standard deviation) for players in each of the competitive CA groups. Three players in the total sample were evaluated as skeletally mature, including the player 16.4 years, and were simply noted as mature and an SA is not assigned. All statistical analyses were done using IBM SPSS (version 24, 2016).

## Results

Descriptive statistics for the total sample of 61 adolescent table tennis players are summarized in Table 1. CAs for the total sample ranged 9.03–16.43 years, while the players were equally distributed by birth trimester. Mean CA was 12.4 years, while mean SA was 13.2 years. Excluding the 3 skeletally mature players, the distribution of participants by skeletal maturity status was as follows: one late (SA-CA<-1.0 years), 32 average (-1.0 < SA-CA < + 1.0 years), and 25 early (SA-CA > 1.0 years). Corresponding means and standard deviations for the 60 players in the three competitive CA groups, U11 (*n* = 13), U13 (*n* = 26) and U15 (*n* = 21), are presented in Table 2. Mean estimates of relative fat mass fluctuated within a narrow range among the three competitive CA groups, respectively, 21.0%, 20.5% and 22.8%. In contrast, height and weight increased linearly with CA, respectively, + 7.6 cm and + 7.1 kg between U11 and U13, and 7.1 cm and 8.3 kg between U13 and U15.

**Table 1:** Descriptive statistics (means and standard deviations) for the total sample of female adolescent table tennis players (*n* = 61)

Variable	units	f	range		Mean		Standard deviation
			min	max	value	(95%CI)	
Training experience	years		1	8	3.2	(2.7 to 3.6)	1.8
Chronological age	years		9.0	16.4	12.4	(12.0 to 12.8)	1.5
Birth quarters							
January - March		15					
April - June		15					
July - September		18					
October – December		13					
Skeletal age^a^	years		9.7	17.6	13.2	(12.7 to 13.6)	1.7
SA standard error^a^	years		0.3	0.6	0.3	(0.29 to 0.33)	0.06
SA-CAa	years		-1.3	3.5	0.9	(0.7 to 1.2)	1.0
Skeletal maturity status							
Late		1					
On time		32					
Early		25					
Mature		3					
Stature	cm		133.0	176.4	157.5	(155.1 to 159.8)	9.2
Body mass	kg		26.2	76.0	48.6	(45.6 to 51.6)	11.5
Skinfold triceps	mm		3	28	13.5	(12.1 to 14.9)	5.4
Skinfold subscapular	mm		4	33	11.0	(9.2 to 12.6)	6.5
Fat mass	%		8.4	40.3	21.3	(19.4 to 23.1)	7.3
	kg		2.2	25.0	10.9	(9.4 to 12.4)	5.7
Fat free mass	kg		22.5	55.3	37.7	(35.8 to 39.5)	7.2

*f* frequency, *SA* skeletal age, *CA* chronological age, 95% *CI* 95% confidence interval

^a^Three skeletally mature players were not considered in the analysis

**Table 2:** Means and standard deviations for the characteristics of female table tennis players (*n* = 60)^†^ by competitive chronological age (CA) groups

Variable	units	Competitive CA Groups		
		U-11 (*n* = 13)	U-13 (*n* = 26)	U-15 (*n* = 21)
Training experience	years	2.2±1.2	2.6±1.1	4.2±1.9
Chronological age	years	10.4±0.6	12.1±0.6	13.8±0.5
Skeletal age	years	11.3±0.9	13.0±1.0	14.8±1.4^‡^
SA standard error	years	0.27±0.01	0.29±0.04	0.36±0.08^‡^
SA-CA	years	0.9±0.8	0.9±0.9	1.0±1.3^‡^
Skeletal maturity				
Late	f	0	0	1
On time	f	8	14	10
Early	f	5	12	8
Mature	f	0	0	2
Stature	cm	148.9±8.7	156.5±8.3	163.5±6.3
Body mass	kg	40.1±11.1	47.2±10.3	54.8±9.7
Triceps skinfold	mm	13.3±5.6	13.3±5.6	14.1±5.3
Subscapular skinfold	mm	11.2±9.4	10.0±5.9	11.4±5.1
Fat mass	kg	9.2±6.4	10.2±5.5	12.5±5.3
	%	21.0±9.4	20.5±7.3	22.2±6.2
Fat free mass	kg	30.9±6.0	37.0±6.0	42.3±5.9

*f* frequency, *SA* skeletal age, *CA* chronological age

^†^The player16.4 years of age was not included in the analysis of competitive CA groups

^‡^Two skeletally mature U15 players were not considered in the calculations of SA and SA-CA

**Fig. 1:**
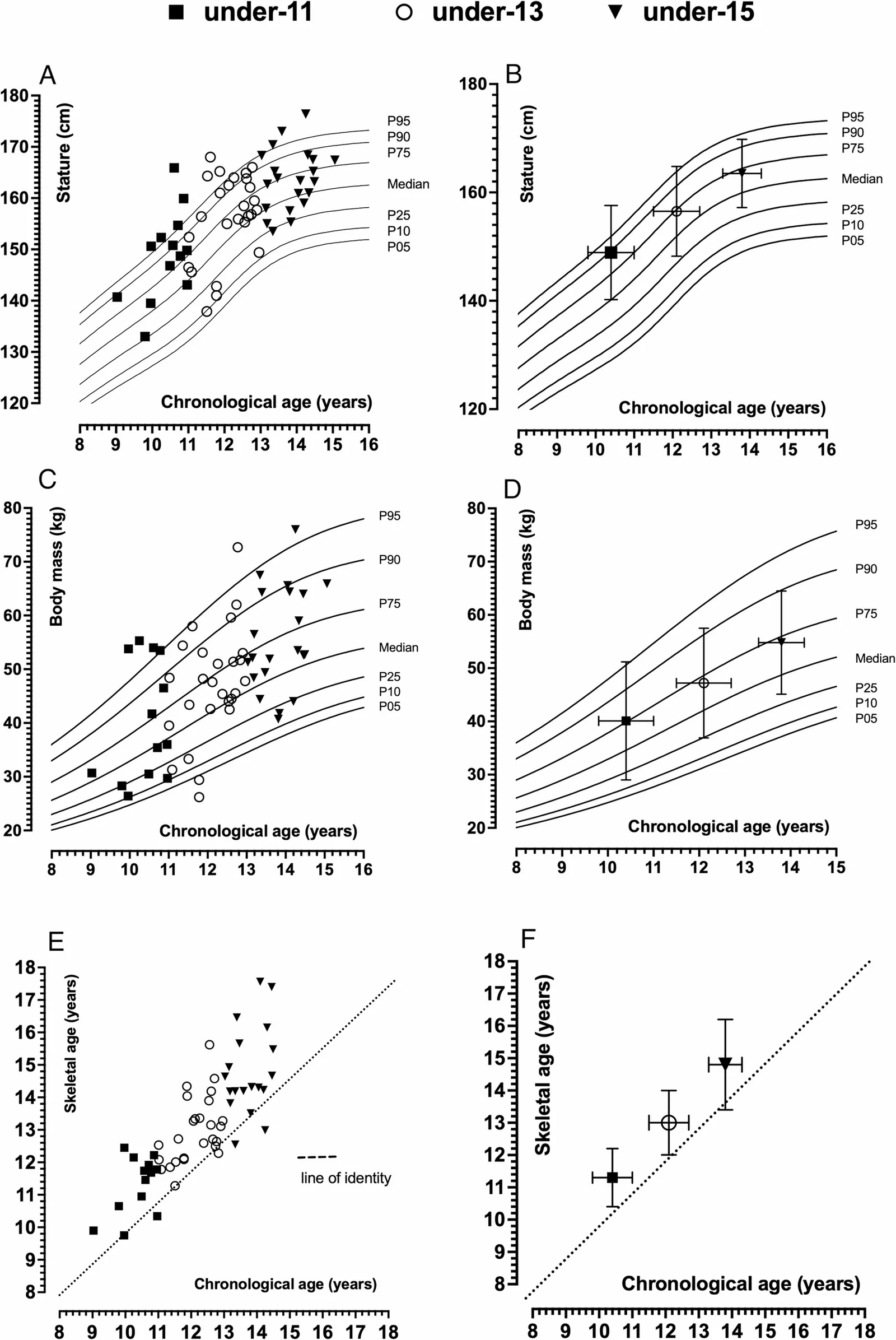
Panel** A**: Statures of individual female table tennis players (*n* = 60) relative to selected percentiles of the reference for U.S. girls [19] according to competitive CA groups; Panel** B**: Mean statures of female table tennis players (*n* = 60) by CA groups relative to selected percentiles of the reference for U.S. girls [19]; Panel** C**: Body masses of individual female table tennis players (*n* = 60) relative to selected percentiles of the reference for U.S. girls [19]; Panel** D**: Mean body masses of female table tennis players (*n* = 60) by CA groups relative to selected percentiles of the reference for U.S. girls [19]; Panel** E**: Skeletal ages of individual female table tennis players (*n* = 58) relative to their chronological ages - the solid line is the line of identity (SA = CA); Panel** F**: Mean skeletal ages of female table tennis players (*n* = 58) relative to mean chronological ages by chronological age group - the solid line is the line of identity (SA = CA)

The statures and weights of individual table tennis players (*n* = 61) are plotted relative to the U.S. reference charts for U.S. girls in, respectively, Fig. 1A and C, while the corresponding means for the three competitive CA groups are plotted in, respectively, Fig. 1B and D. For both indicators of body size, variation among players is substantial with several players at or below the 10th percentile and others at or above the 90th percentile. The means for stature and body mass of the players by competitive CA groups approximate the 75th percentile for the general population of U.S. girls. Consistent with the larger body size, SAs of individual players (excluding the skeletally mature players) are largely in advance of their respective CAs (Fig. 1E), and mean SAs for players in each competitive CA group are, on average, in advance of the respective mean CAs (Fig. 1F), highlighting the advanced skeletal maturity status of the sample.

## Discussion

Results of this study of a select sample of youth female table tennis players indicated considerable inter-individual variability in growth status with statures and weights spanning the 10th to above the 90th percentiles of the U.S. reference for girls. The mean statures and weights of the three competitive CA groups were also above the respective reference medians for the general population. The trend in body size was consistent with the advanced skeletal maturity status of the sample. Although the total sample of players ranged from 9.0 to 16.4 years in CA, SAs ranged 9.7 to 17.6 years, and the mean SA in each competitive CA group exceeded the corresponding mean CA. Overall, mean SA for the total sample (13.2 ± 1.7 years) exceeded mean CA (12.4 ± 1.5 years) by about 0.8 year. The standard deviation for SA was larger than the corresponding standard deviations in CA in each competitive CA group.

Except for a study of the growth and skeletal maturity status of youth male table tennis players 8.4–15.3 years [6], corresponding studies of youth participants of both sexes in the sport are lacking. As in the current study of female participants in table tennis, the stature and body mass of male participants spanned the corresponding 10th and 90th percentiles of the US reference, and the age-specific mean statures of players 10 through13 years fluctuated between the 75th and 90th percentiles of the reference, while at 14 years of age the statures and weights of the players tended to approximate the reference medians; and also, as among female participants, mean SAs of males were consistently in advance of CA [6]. Similar trends were noted for the skeletal maturity status, also assessed with the Fels method, of 44 elite British female tennis players 8–17 years [7]. Among the British players, 29 of 44 were average in skeletal maturity status with two participants classified as delayed and 13 as advanced. In the current study, only one female adolescent table tennis player was delayed, while 32 were average and 25 were advanced in skeletal maturity status (Table 1). Only three table tennis participants in the current sample were skeletally mature and not included in the analysis. Although the number of participants is relatively limited, the range of variability in SA within and among competitive CA groups in youth sports is relevant.

Allowing for variation among methods of assessment, SA tends to be advanced relative to CA among adolescent participants in a variety of sports with unique demands as in, for example, soccer [20], roller hockey [21], tennis [7] and table tennis [6]. Several recent studies have also examined data quality inherent to SA assessments with different methods [22–24]. For example, intra-observer reproducibility of assessments of maturity indicators and assigned SAs using the Fels method was considered for two examiners in a sample of male tennis players 8.7–16.8 years of age [23]. Among trained examiners, SA assessments were highly reproducible, although not perfect; distributions of players by maturity status were also similar in the two examiners. Based on the frequencies of errors, the following specific indicators in the hand-wrist skeleton emerged as most problematic: metacarpal III (proximo-medial projection of the epiphysis), trapezoid (shape of the medial margin), triquetral (shape of the lateral margin), and trapezium (radiopaque line or zone within the proximal margin).

The literature has also considered different protocols for the assessment of SA in the same players. For example, among 40 elite male Spanish youth soccer players 12.5–16.1 years, TW3 and Fels methods yielded different SAs, and significantly more players were classified as skeletally mature with the TW3 method than with the Fels method, particularly after 14 years of age [25]. The latter likely reflects the criterion for the final stage of, respectively, the radius and ulna with the TW method; it is simply “Fusion of the epiphysis and metaphysis has begun” [26]. The interval between initiation of fusion and complete epiphyseal union is not considered. Note also that TW3 SAs and TW2 SAs based on the radius-ulna-short bone protocol also differed significantly among male soccer players from eight countries in single year CA groups spanning 11 through 17 years of age [27]. TW3 SAs were consistently lower than TW2 SAs across the CA range, although players identified as skeletally mature did not differ as the criterion for the final stages of, respectively, the radius and ulna was the same for TW2 and TW3 RUS SAs.

Allowing for the relatively limited information on female youth participants in racquet sports, the heights and weights of female players in several racquet sports are summarized in, respectively, Tables 3 and 4. The adolescent athletes tend to be characterized by statures and body weights that were, on average, slightly above the respective reference medians. Nevertheless, it is often intuitively assumed that success of youth in table tennis during pubertal years is more dependent on perceptual skills [28]. Of interest and based on sports different from table tennis, sport-specific skills were not associated with skeletal maturity status among youth soccer players [29] and with sexual maturity status among youth basketball players [30].

**Table 3:** Descriptive statistics (mean ± standard deviation) for stature in several studies of adolescent female athletes in table tennis and tennis (racquet sports) and age-specific percentiles of the reference for US girls [19]; the percentiles of the reference and the percentiles approached by the mean values of the table tennis and tennis players is indicated in bold print for each CA group

Study	Sport	Chronological Age		*n*			Population	US Reference Centers for Disease Control and Prevention^a^				
		years	months		Mean	Standard deviation		10	25	50	75	90
present study	table tennis	09.69	116	4	141.0	7.3	Portuguese	128.3	132.1	136.5	**141.0**	145.1
		10.69	128	9	152.4	6.9		133.1	137.3	142.1	146.9	**151.3**
		11.49	138	11	152.8	10.7		137.7	142.2	147.2	**152.2**	**156.7**
		12.58	151	15	159.4	4.6		145.5	149.9	154.8	**159.5**	163.8
		13.39	161	11	162.1	6.7		150.0	154.1	**158.7**	**163.2**	167.3
		14.29	171	9	165.0	5.3		152.4	156.4	160.8	**165.2**	169.2
		15.06	181	1	167.4			153.6	157.5	161.9	**166.3**	**170.2**
		16.43	197	1	165.5			154.4	158.3	**162.7**	**167.1**	171.0
Söğüt et al. (2019)[31]	tennis	11.10	133	51	148.7	9.3	Turkish	135.0	139.4	**144.3**	**149.2**	153.7
Myburgh et al. (2016)[7]	tennis	09.81	118	10	141.7	7.4	British, elite	129,0	132,9	137,3	**141**,**9**	146,0
		11.79	141	17	156.1	6.6		139.5	144.1	149.1	**154.1**	**158.5**
		14.11	169	12	164.0	4.5		152.1	156.0	160.5	**164.9**	168.9
		15.67	188	5	166.3	8.9		154.1	158.0	162.3	**166.7**	170.7
Kramer et al. (2021)[32]	tennis	10.30	124	15	145.8	6.0	Dutch, elite	131.3	135.4	140.1	**144.8**	**149.1**
		11.04	132	40	150.8	6.1		134.0	138.3	143.1	**148.0**	**152.5**
		11.98	144	43	156.4	5.7		141.3	145.9	150.9	**155.9**	**160.3**
		12.95	155	50	163.5	4.4		147.5	151.8	156.6	**161.3**	**165.4**
		13.94	167	37	167.7	4.4		151.6	155.6	160.1	**164.6**	**168.6**
		14.82	178	16	169.3	3.8		153.3	157.3	161.6	**166.0**	**170.0**

^a^Mean values obtained from studies approximate the percentiles indicated in bold

**Table 4:** Descriptive statistics (mean ± standard deviation) for body mass in several studies of adolescent female athletes in table tennis and tennis (racquet sports) and age-specific percentiles of the reference for US girls [19]; the percentiles of the reference and the percentiles approached by the mean values of the table tennis and tennis players is indicated in bold print for each CA group

Study	Sport	Chronological Age		*n*			Population	US Reference Centers for Disease Control and Prevention^a^				
		years	months		Mean	Standard Deviation		10	25	50	75	90
present study	table tennis	09.69	116	4	34.8	12.8	Portuguese	25.4	28.0	**31.7**	**36.4**	41.9
		10.69	128	9	42.5	10.2		28.4	31.6	35.9	**41.4**	**47.9**
		11.49	138	11	42.3	11.0		31.3	34.8	**39.6**	**45.8**	53.0
		12.58	151	15	50.8	8.4		34.9	38.7	44.0	**50.8**	58.7
		13.39	161	11	51.7	8.5		37.8	41.7	**47.2**	**54.4**	62.9
		14.29	171	9	59.1	9.5		40.5	44.4	50.0	**57.3**	**66.2**
		15.06	181	1	65.9			42.8	46.7	52.1	**59.4**	**68.5**
		16.43	197	1	52.5			45.4	**49.1**	**54.4**	61.6	70.9
Söğüt et al. (2019)[31]	tennis	11.10	133	51	41.6	9.8	Turkish	29.5	32.9	**37.4**	**43.2**	49.9
Myburgh et al. (2016) [7]	tennis	09.81	118	10	33.2	5.5	British, elite	25.6	28.3	**32.0**	**36.8**	42.4
		11.79	141	17	44.5	5.6		31.9	35.4	**40.4**	**46.6**	54.0
		14.11	169	12	57.0	6.5		40.0	43.9	49.5	**56.8**	65.6
		15.67	188	5	61.3	7.7		44.1	47.9	53.3	**60.5**	**69.7**
Kramer et al. (2021)[32]	tennis	10.30	124	15	34.4	4.0	Dutch, elite	27.1	30.1	**34.1**	39.3	45.4
		11.04	132	40	38.2	4.7		29.3	32.5	**37.0**	**42.8**	49.4
		11.98	144	43	41.9	4.4		32.8	36.4	**41.5**	47.9	55.5
		12.95	155	50	50.1	4.8		36.0	39.9	**45.3**	**52.3**	60.5
		13.94	167	37	55.3	4.1		39.4	43.4	**49.0**	**56.2**	65.0
		14.82	178	16	59.0	3.8		42.1	46.0	51.6	**58.9**	67.9

^a^Mean values obtained from studies approximate the percentiles indicated in bold

Anthropometric, maturity and functional characteristics of youth athletes are often considered in the context of talent identification and selection for a sport, and relative to the incidence of injuries, more so among males than females. In the context of youth table tennis, each of the three competitive CA groups considered in the present study (U11, U13, U15) span two years. Nevertheless, variation within the two-year CA groups may limit opportunities to experience success among younger players within the respective competitive categories. Grouping players by biological maturity status has been suggested in some talent development models, including the Long Term Athlete Development model [33]. Although the present study was limited to female players through the U15 category, two participants were classified as skeletally mature with the Fels method (as well as the one player 16.4 years) and given the relatively small number of female participants in many competitive settings, a more condensed CA category structure may merit consideration. This observation also suggests that the growth and maturity characteristics of female players in the U17 category warrant further study. Nevertheless, interactions among growth and maturity status and performance characteristics during the interval of the adolescent growth spurt merit closer attention in the context of the demands of specific sports, and also of perceptions of self-efficacy and self-concept.

The present study is not without limitations. Although the study highlights variation in the growth and biological maturity status of female youth table tennis players, the sample is limited to Portuguese players who requested to train and compete in older competitive CA groups. There is a need for studies of the growth and maturity characteristics of a larger sample of female table tennis players competing at local, national and international levels. And as already reported for other sports, longitudinal developmental studies reflecting inter-individual and intra-individual variation in functional capacities and sport-specific skills are also needed in youth table tennis and perhaps other sports.

## Conclusions

The present study considered a specific sample of female youth table tennis players aged 9.0–16.4 years who underwent medical evaluation to obtain permission for competing in older CA categories. The interpretation of body size and body composition during adolescence should not be dissociated from the SA assessments. The variation in skeletal maturity status also supports consideration of female specific competitive CA categories that differ from those of male youth table tennis players. Nevertheless, the current findings should be interpreted with caution when generalizing to the broader population of youth female table tennis players. Caution is also needed when extrapolating the observations to other racquet sports. The substantial individual variation in growth, skeletal maturity status, and body composition observed among the players supports the need for future research combining SA assessments with complementary indicators of maturity timing, specifically age at menarche and age at peak height velocity.

## Supplementary Information

Supplementary Material 1.

## Data Availability

Data is provided within the manuscript or supplementary information files.
